# Clinicopathologic and immunohistochemical characterization of 14 cases of angioleiomyomas in oral cavity

**DOI:** 10.4317/medoral.22649

**Published:** 2018-09-28

**Authors:** Juan Aitken-Saavedra, Karine-Duarte da Silva, Ana-Paula-Neutzling Gomes, Ana-Carolina-Uchoa Vasconcelos, Adriana Etges, Thaíse-Gomes Nóbrega, Sandra-Beatriz-Chaves Tarquinio

**Affiliations:** 1 Center of Diagnosis of Diseases of the Mouth, Federal University of Pelotas. Pelotas, Brazil; 2 Post Graduate program in dentistry. Federal University of Pelotas. Pelotas, Brazil; 3 Department of Oral Pathology and Medicine, School of Dentistry, University of Chile, Santiago, Chile

## Abstract

**Background:**

Angioleiomyoma (ALM) is a benign neoplasm that originates from vascular smooth muscle. It is extremely rare in oral cavity. The objective of this study was to evaluate the clinicopathological and immunohistochemical characteristics of all oral angioleiomyomas registered in a Center of Diagnosis of Oral Diseases from 1959 to 2017.

**Material and Methods:**

Slides from 14 cases of ALM stained with hematoxylin and eosin (H&E) were analyzed to confirm the diagnosis. Moreover, an immunohistochemical panel with alpha-smooth muscle actin (alpha-SMA), desmin, AE1/AE3, CD68, S-100, and CD34 antibodies was performed to evaluate semi-quantitatively the positive cells.

**Results:**

ALM correspond to 0.08% of all benign oral tumors analyzed during the 57-year period. The mean age of the patients was 45 years with a predilection to males (58%). The most frequently reported site was lips (50%). Microscopic analysis on H&E sections revealed similar pattern in all cases, showing well-circumscribed and encapsulated tumors, characterized by a proliferation of smooth muscle cells and wide vascular spaces of varying sizes. The predominant immuno profiles were: alpha-smooth muscle actin (alpha-SMA) positive (strong immunoreactivity); positive variable pattern for desmin, negative immunoprofile for AE1/AE3, CD68, and S-100. The endothelial cells of vascular spaces were CD34+.

**Conclusions:**

Based on the results, the alpha-SM actin can be elected as a good marker for angioleiomyomas and can help the confirmation of the morphologic diagnosis of this lesion.

** Key words:**Angioleiomyoma, Alpha-SMA, vascular smooth muscle.

## Introduction

Angioleiomyoma (ALM) is a neoplastic proliferation of vascular smooth muscle cells (1). Most authors accept that ALM originates from smooth muscle in the walls of vascular channels (2), others have suggested that ALM corresponds to a kind of hamartoma (3), a vascular malformation (4), or one stage in a continuous process of smooth muscle proliferation from hemangioma to solid leiomyoma. They are extremely rare in oral cavity, reaching a value of 0.065% (5-7), due to the paucity of smooth muscle in this site where the tunica media of blood vessels is the primary source of smooth muscle. Microscopic analysis shows well-circumscribed and encapsulated tumor characterized by proliferation of smooth muscle cells and vascular spaces of varying sizes (Ide, 2004). It was proposed a classification system which divided ALM into three histological subtypes (solid, cavernous, and venous) (2). In establishing a definitive diagnosis of angioleiomyoma, the use of a immunohistochemical panel is recommended, in addition to conventional hematoxylin and eosin (H&E) staining (8). The last review in this topic was published in 2014 and it reported less than 200 cases of angioleiomyomas in the head and neck area (9). The present study retrospectively examined the clinical and histological characteristics of all oral ALM which were diagnosed in a reference center, from 1959 until 2017. To the best of our knowledge, this is the first study of this neoplasia in which, besides H&E staining, all the samples were subjected to immunohistochemistry, using a panel of antibodies to investigate them, such as alpha-smooth muscle actin (alpha-SMA), desmin, AE1/AE3, CD68, S-100, and CD34 antibodies, aiming to make a definitive diagnosis. Besides being the first report made in Latin America, there is no other report that has evaluated the oral angioleiomyomas in a population for such an extensive period of time. The objective of this study was to evaluate the clinicopathological and immunohistochemical characteristics of all oral angioleiomyomas registered in a Center of Diagnosis of Oral Diseases from 1959 to 2017.

## Material and Methods

Fourteen cases of ALM were retrieved from the Center of Diagnosis of Oral Diseases of the School of Dentistry/Federal University of Pelotas, over a period of 57 years (1959 to 2017). The analyzed variables included sex, age, and lesion location. H&E-stained slides of these lesions were evaluated to confirm the diagnosis of ALM. In addition, the tumors were classified according to the criteria proposed by Morimoto (2) (solid, venous, and cavernous). Moreover, in order to better establish the diagnosis, the samples were submitted to an immunohistochemical panel of antibodies to distinguish mesenquimal and epithelial cells. The study followed the recommendations of the Declaration of Helsinki for medical protocol (10) and was approved by the Ethical Review Board of the School of Dentistry of the Federal University of Pelotas.

-Immunohistochemistry

Tissues were submitted to immunohistochemical technique using the antibodies for alpha-smooth muscle actin (alpha-SMA), desmin, AE1/AE3, CD68, S-100, and CD34. Sections were deparaffinized in xylol and hydrated in a decreasing ethanol solution. Antigen retrieval was performed with a TRIS-EDTA solution (pH 9.0) in a 96 °C water bath for 30 min. The hydrogen peroxide blocking, protein blocking, and detection steps were performed with ready-to-use solutions provided in the kit (Spring BioScience, SPB-999). The reaction was revealed with 3,3′-diaminobenzidine (Spring BioScience, code DAB- 999) and was counterstained with Harris’ hematoxylin. Appropriate positive and negative controls were included.

-Evaluation of immunohistochemistry

One observer evaluated all cases using an optical microscope. A semi-quantitative analysis of positive cells was made. Five high-power fields (400× magnification) were analyzed separately for each immunohistochemical reaction in the intratumoral region and positivity was graded as – (no staining observed), + (1%–25% of positive cells), ++ (26%–50%), +++ (>50%), according to the studies performed by Lundqvist *et al.* (11), Shinriki *et al.* (12) , and Caldeira *et al.* (13) .

-Statistical analysis

Data collected were typed in the Microsoft Excel® and the statistical tests were performed using STATA 12 ®. The results were described descriptively.

## Results

Distribution: The patients diagnosed with angioleiomyomas accounted for 0.08% (21 out of 24,600) of all benign oral tumors analyzed histopathologically during the 57-year operating period of Center of Diagnosis of Oral Diseases.

Clinical and histopathological features: The variables of sex, age and lesion location for the analyzed cases are shown in  Table 1. The diagnosis of angioleiomyoma was more common in males (n = 8, 57.1%) with a mean age of 45.2 years. Nine cases (64.3%) were diagnosed between fourth to sixth decades of life. Seven cases (about 50%) were detected in lips (upper and lower lip), followed by buccal mucosa (4%–28.4%), soft palate (2%–4.3%), and hard palate (1%–7.1%). All tumors were solitary and well-circumscribed. In relation to the classification system of Morimoto (Wang *et al.*, 2004), eight cases (57%) were solid type tumors, four (28%) were cavernous and two (14%) were venous.

**Table 1:**
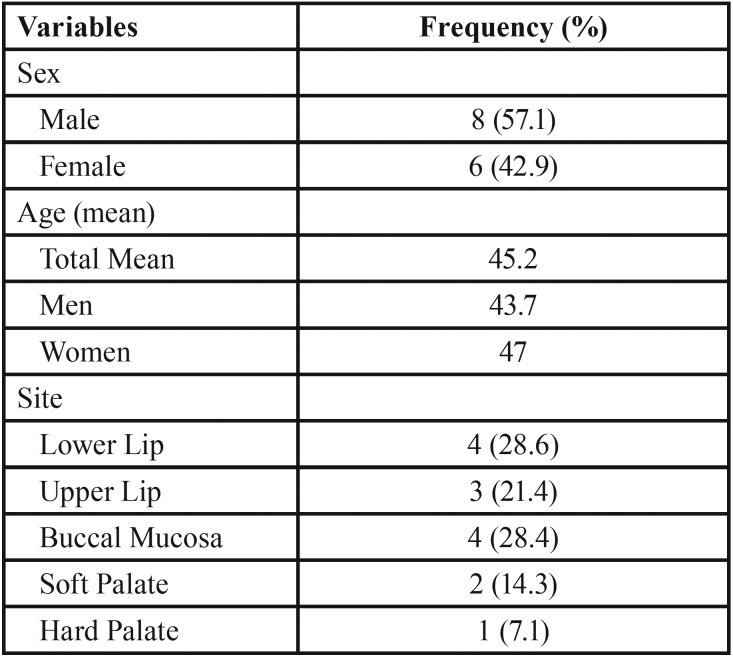
Characteristics of the sample (n = 14). Pelotas, Brazil.

**Figure 1:**
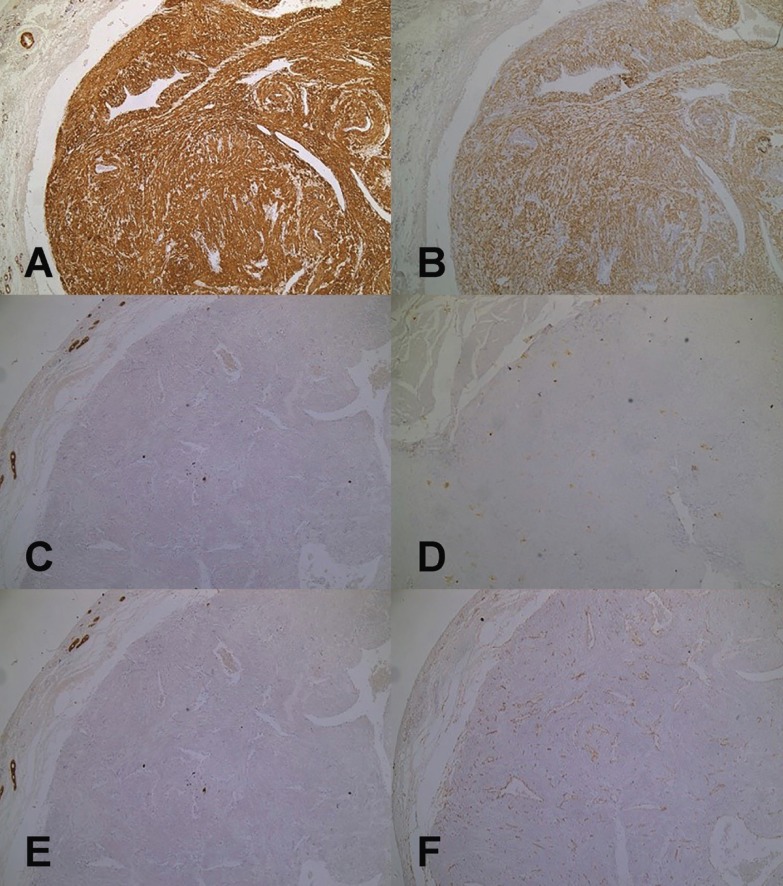
Immunohistochemical characteristics of the oral angioleiomiomas (n = 14). Pelotas, Brazil. A) Positive and strong immunoreaction for alpha- smooth muscle actin antibody showed by neoplastic cells. (IHC, X4). B) Positive and variable immunoreaction for desmin antibody showed by neoplastic cells. (IHC, X4). C) Negative immunoreaction for CD68 antibody showed by neoplastic cells. (IHC, X4). D) Negative immunoreaction for S100 antibody showed by neoplastic cells. (IHC, X4). E) Negative immunoreaction for AE1/AE3 antibody showed by neoplastic cells. (IHC, X4). F) Positive immunoreaction for CD34 antibody showed by the endothelial cells lining the vascular spaces. (IHC, X100).

Immunohistochemistry: In all cases a strong immunoreactivity for α-smooth muscle actin was observed in the cellular membrane of the neoplastic cells, being the immunopositivity for desmin variable among the tumors. The samples also showed negativity for the antibodies S-100, AE1/AE3, and CD68. The endothelial cells of vascular spaces were positive for CD34 antigen ( Table 2 and Fig. 1).

**Table 2:**
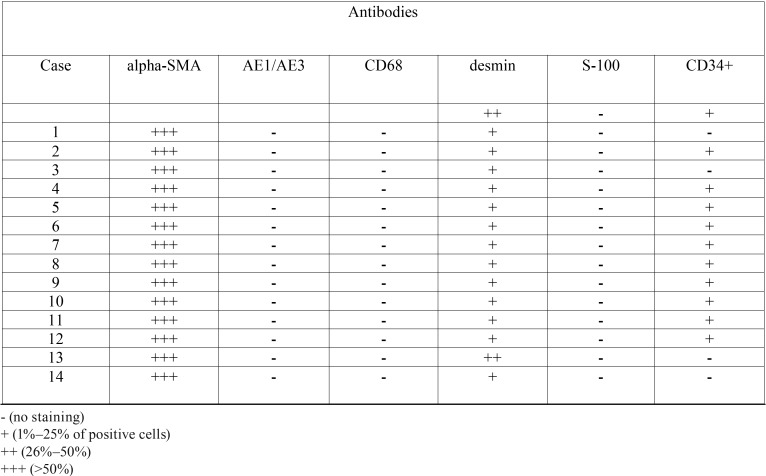
Immunostaining of oral angioleiomyomas to alpha-smooth muscle actin (alpha-SMA), AE1/AE3, CD68, desmin, S-100, and CD34+ antibodies. Pelotas, Brazil.

## Discussion

Angioleiomyomas are benign soft tissue tumors that originate from vascular smooth muscle and are extremely rare in the head and neck. An evaluation of their immunohistochemical pattern and a detailed analysis of their histomorphology are recommended for their diagnosis. The present study examined the characteristics of oral angioleiomiomas diagnosed in the Center of Diagnosis of Oral Diseases of the School of Dentistry of the Federal University of Pelotas from 1959 to 2017. A complete immunohistochemical panel was performed for all the samples in addition to the conventional H&E staining to make a definitive diagnosis.

Epidemiological data about ALM are scarce. Liu *et al.* (9) showed that the frequency of these lesions in a pathological service (Chengdu, Sichuan, China) represented 0.18% of all benign head and neck tumors. In the oral cavity, they are still considered rare. Baden (5) describes only five out of 7,748 (0.064%) oral ALM cases in a series of benign tumors in all body sites, showing similar results as those of the present analysis, which revealed only 14 out of 24,600 cases (0.08%) in a reference service, during more than 40 years.

Regarding the age of occurrence of oral ALM, our results are similar to those previously described in the few epidemiological reports made in this regard. Our cases of oral ALM averaged a presentation age of 45.2 years. The average age reported by similar studies for the same tumor are 48 (9) ,42.5 (1) ,45 (9) and 47.4 (14) years. In the same way, Gaitan-Cepeda *et al.* (8) reported highest prevalence of oral ALM in the fourth and fifth decades of life. In our study, eight (57.1%) of the patients were men. Articles generally describe a slight tendency of oral AML to occur in men. Wang et al. 2 reports a frequency identical to ours in this sex (57%), and Yoon *et al.* (15) reported a 75% frequency in men. Brooks *et al.* (1) describes a predilection of male to female in a proportion of 1.43:1, and Liu (9) relates a proportion of 1.625:1 also with a predilection for men. Gueiros *et al.* (14) showed that 62,5% of the cases affected males, yielding a male to female ratio of 1,67:1. Regarding the location of oral ALM, a Chinese reference center reported 21 cases in 34 years, the most affected sites being buccal mucosa, parotid, and palate, which differs from the present study since 49.7% of the cases were described in lips (28.3% in lower lip and 21.4% in upper lip). However, similar to this analysis, Brooks *et al.* (1) reported in a study carried out for 38 years in India that the lip was the most affected site (48.6%) for ALM, followed by palate (21.1%).

It was proposed a classification system which divided AML into solid, cavernous, and venous type (2) and reported that these pathologic subtypes were related to different clinical manifestations. According to this classification system, in our study, eight cases (57%) were of the solid type, four (28%) were of the cavernous type, and two (14%) were of the venous type. Our results are relatively similar to those described in Taipei, Taiwan, where 14 cases (67%) were solid, six (28%) were cavernous, and six (5%) were venous (9). Another study made in Chengdu, China indicated that 12 oral ALMs (42%) were of the solid type, four (33%) were of the venous type, and three were (25%) of the cavernous type (9). Nevertheless, in our study and in the above-mentioned studies, the histologic subtype was not related to sex, lesion location, and presence of symptoms. In general, similar studies are not conclusive about the relationship between histological subtypes and differences in treatment, prognosis, or symptomatology.

Angioleiomyomas are difficult to diagnose correctly only from clinical manifestations and imaging 1, and they seldom are included in the differential diagnosis of an oral tumor before surgical excision. After that, although most angioleiomiomas can be correctly diagnosed under microscopy with conventional H&E staining, it is recommended to use an antibody panel to confirm the diagnosis and differentiate it from other tumors. It is important to differentiate angioleiomyoma from other types of spindle cell tumor, including leiomyoma (CD34- and S-100-), myopericytoma (desmin-, CD34-, and S100-), and myofibroma (desmin-, CD34-, and S-100-/+) (8). It is particularly important to distinguish ALM from malignant mesenchymal tumors, including leiomyosarcoma, in which pleomorphism and cellular proliferation (ki-67) are more evident (16). As each of the mentioned lesions presents a different nature, which determines the prognosis and treatment of the affected patients, making these differential diagnoses is important. In our study, immunohistochemical staining was highly positive for alpha-SMA and partially positive for desmin. Actin is found in eukaryotic cells and constitutes one of the most abundant cellular proteins available. There are about six actin isoforms and alpha-SMA is considered almost specific for smooth muscle tissue (17,18). Positive expression of CD34 demonstrated the presence of vascular endothelium. In our study, no instances of immunohistochemical staining were related to the histological subtype, age, sex, lesion location, or symptomatology of angioleiomiomas.

Based on our results, only alpha-SMA can be elected as a good marker for angioleiomyomas and can help the confirmation of the morphologic diagnosis of this lesion.
